# Engagement communautaire et prise en compte de la détresse psychologique, de la peur et de la stigmatisation dans la surveillance et la gestion des épidémies de la maladie à virus Ebola dans l’approche « Une seule santé » en République démocratique du Congo

**DOI:** 10.1177/17579759241277117

**Published:** 2024-10-17

**Authors:** Dieudonné Kazadi Mwamba, Christina Zarowsky, Célé Depaul Manianga, Serge Kapanga, Grégory Moullec

**Affiliations:** 1 Université de Montréal, Montréal, QC, Canada; 2 Université de Kinshasa, Kinshasa, Congo

**Keywords:** stress, communautés, santé mentale, résilience, action communautaire, maladies transmissibles

## Abstract

**Introduction et objectif:**

Une réponse efficace aux crises sanitaires telle la maladie à virus Ebola (MVE) nécessite l’engagement des communautés. Cette étude explore les enjeux psychosociaux influant sur la réponse des communautés congolaises aux épidémies de MVE, et comment la détresse et la résilience communautaire s’intègrent dans l’approche « Une seule santé » selon les perceptions des communautés.

**Méthodes:**

Cette étude de cas qualitative au nord Kivu inclut des entretiens individuels (*n* = 36) avec des informateurs clés et trois groupes de discussions avec des participants (*n* = 28) issus de diverses associations locales. Le cadre MATCH de l’engagement communautaire, adapté, structure l’analyse thématique.

**Résultats:**

Les participants ont tous reconnu le vécu douloureux – la peur, le rejet, la stigmatisation – des communautés et des professionnels de santé, et le rôle crucial des communautés dans la réponse aux épidémies. Ils ont souligné l’importance d’impliquer ces communautés dans l’élaboration et la mise en œuvre des stratégies pour éviter l’« Ebola business ». Les perceptions face à la maladie des différentes catégories de participants sont influencées par des croyances, les rôles sociaux et des considérations religieuses. Concernant l’approche « Une seule santé », les participants ont reconnu la nécessité d’impliquer toutes les couches sociales de la communauté.

**Conclusion:**

Un modèle innovant de gestion des épidémies et urgences de santé publique intègre les essentiels de l’engagement communautaire dont la détresse psychologique.

## Introduction

L’engagement des communautés dans la gestion des crises, en particulier de la maladie à virus Ebola (MVE), est un élément incontournable pour une réponse efficace à la crise (1). L’engagement communautaire et la prise en compte de la détresse psychologique sont des facteurs clés pour une meilleure assistance à la population et une humanisation de la prise en charge. Cette étude explore le rôle des communautés dans la gestion des épidémies de MVE, notamment les perceptions et les aspects psychosociaux intervenant dans la réponse.

La notion de « communauté » est complexe. Elle peut être différemment comprise selon différentes catégories de personnes et dans des contextes ou des situations différentes (2). Les communautés sont définies par leur culture, environnement, croyances, idéologies, activités, responsabilités sociales et sentiment d’appartenance à un groupe (3,4). Face à des épidémies, elles sont confrontées à des défis uniques, influencées par leurs pratiques et par leur perception de la maladie – y compris les interprétations culturelles de la causalité des maladies (5). Le recours aux soins traditionnels prime souvent sur les formations sanitaires vues comme une voie directe vers le décès (6). La famille demeure le noyau social au sein duquel le malade trouve son soutien. La proximité géographique et le quartier jouent un rôle épidémiologique important lors des éclosions et facilitent aussi l’accompagne-ment du patient (7). Dans cet article, nous nous penchons surtout sur les communautés géographiques et culturelles, sans oublier les rôles importants des réseaux sociaux et de l’information qui dépassent les limites d’un village, d’un quartier ou d’une ville.

### Défis spécifiques des épidémies de MVE

Dans les épidémies de MVE, les communautés locales paient toujours un lourd tribut avec une mortalité importante, y compris du personnel et des femmes, Ces dernières jouant, dans les cultures africaines, un rôle d’assistance et de soutien aux malades (8). Les comportements socioculturels comme les pratiques funéraires, la négation de la maladie et la non-observance des mesures de prévention amplifient souvent le risque de propagation de la maladie (8,9). Pourtant, les enquêtes menées sur les connaissances, les attitudes et les pratiques des populations en rapport avec la MVE ont montré qu’une majeure partie de la population, soit 92 % (10,11), connait les mesures de prévention, les modes de transmission et les causes de la maladie malgré la persistance de fausses idées (12).

Par contre, certains mécanismes d’adaptation ou de résilience ont été développés par les personnes guéries de la MVE pour faire face à ces crises notamment en adaptant leurs activités par la mobilisation de ressources propres aux communautés (13).

### Intérêts d’une approche *Une seule santé*

Au-delà des crises épidémiques, la MVE, en tant que maladie zoonotique (8), illustre la nécessité d’une approche intégrée de la santé. L’approche « Une seule santé » met en avant la connexion entre la santé humaine, animale et de l’environnement. Cette perspective est d’autant plus cruciale dans un contexte où les communautés sont en première ligne face à la maladie et à ses impacts psychosociaux. Elles sont également au cœur des interactions entre humains, animaux et environnement, interactions qui peuvent engendrer ou exacerber des épidémies. Et avec la croissance des populations tant humaine qu’animale, et suite aux changements environnementaux rapides, les liens entre la santé humaine, la santé animale et l’environnement deviennent de plus en plus évidents (14). L’approche « Une seule santé » favorise une collaboration étroite entre les services de santé humaine, animale et environnementale pour la surveillance des maladies zoonotiques.

Mais pour que cette collaboration soit efficace, il est important d’intégrer les dimensions de détresse, souffrance, et résilience communautaire. En reconnaissant l’interdépendance entre ces facteurs, nous pourrons élaborer des réponses plus holistiques et efficaces face aux épidémies.

L’objectif dans cette étude est d’examiner les enjeux psychosociaux qui influent sur la réponse des communautés aux épidémies de MVE, et comment la détresse et la résilience communautaire s’intègrent dans l’approche « Une seule santé ».

### Cadre conceptuel

**Figure 1.**
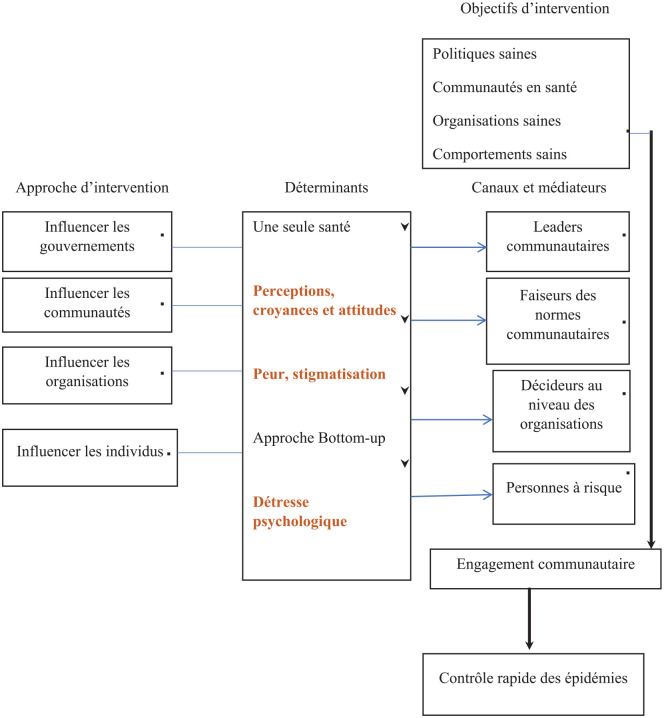
Modèle conceptuel MATCH adapté.

Les éléments essentiels de notre étude (15) (voir Figure 1) sont tirés et adaptés du modèle conceptuel de Simmons, en y intégrant les déterminants majeurs qui influencent l’engagement des communautés, notamment les perceptions, la peur, la stigmatisation, la détresse psychologique et Une seule santé. Les perceptions, la stigmatisation et la détresse psychologique et une Seule santé sont les déterminants pris en compte dans cette étude.

## Matériels et méthodes

### Approbation éthique et consentement

Les approbations éthiques ont été obtenues de l’Université de Montréal et de l’Université de Kinshasa. Nous avons délibérément choisi de ne pas collecter personnellement les données sur le terrain pour éviter d’influencer les répondants étant donné le positionnement du premier auteur (DM), médecin gestionnaire au ministère de la Santé, expert et coordonnateur de la riposte à la MVE. Nous avons eu recours à un assistant de recherche, anthropologue de formation, avec une expérience avérée dans la collecte des données.

Nous avons utilisé la checklist COREQ pour la collecte et le rapportage des données qualitatives (16).

### Type, site et période d’étude

Nous avons mené une étude de cas qualitative sur l’engagement des communautés lors des épidémies d’Ebola survenues au cours de la période 2018 à 2020 dans la province du Nord-Kivu, à l’est de la RDC où nous avons sélectionné deux des zones de santé les plus touchées par la MVE, Goma et Butembo, en raison de leur nombre élevé de cas de MVE pendant l’épidémie. L’étude a eu lieu entre les mois d’octobre et de décembre 2020.

### Échantillonnage

Nous avons interrogé 36 informateurs clés (*n*=36) constitués de jeunes, de femmes, de leaders communautaires, de membres du personnel, et de tradipraticiens choisis de manière raisonnée pour leur expérience dans la gestion de la MVE. Trois groupes de discussion ont aussi été organisés réunissant entre 6 et 10 participants chacun (*n*=28). Les groupes de discussion mixtes ont été réalisés avec des jeunes d’associations ayant participé à la riposte, des étudiants en arts et métiers, et des guéris d’Ebola. Ces groupes comprenaient des hommes et des femmes de différentes professions. Au total, 64 participants ont participé dans l’étude. (Voir Tableau 1. Caractéristiques des participants aux groupes de discussion).

**Tableau 1.** Caractéristiques des participants aux discussions de groupe.

Caractéristiques sociodémographiques	Jeunes	Étudiants	Guéris Ebola	Total
	*n* *=* 22	*n* *=* 20	*n* *=* 22	*n* (%) *=* 64
Genre				
Masculin	12	10	11	33 (51,6)
Féminin	10	10	11	31 (48,4)
Catégorie d’âge (ans)				
18—25 ans	22	20	0	42 (65,6)
26—59 ans	0	0	22	22 (34,4)
60 ans et plus	0	0	0	0 (0)
Niveau d’éducation				
Secondaire	10	0	9	19 (29,7)
Universitaire	12	20	13	45 (70,3)
Post-universitaire	0	0	0	0 (0)
Fonction				
Étudiants	0	12	0	12 (18,8)
Ménagères	3	0	4	7 (10,9)
Infirmiers	0	8	8	16 (25)
Cultivateurs	6	0	5	11 (17,2)
Commerçants	10	0	5	15 (23,4)
Sans emploi	3	0	0	3 (4.7)

### Collecte de données

Tous les entretiens ont été réalisés en français ou en swahili par un expert socio-anthropologue ayant une grande expérience dans les enquêtes qualitatives, avec l’aide d’un interprète. Les enregistrements audios ont été réalisés sur dictaphone, et des notes, y compris des expressions non verbales, ont également été prises lors de chaque entretien, qui durait en moyenne 50 minutes.

### L’analyse des données

Les enregistrements audios en français ont été transcrits, tandis que ceux en swahili ont été traduits vers le français lors de la transcription par deux membres de l’équipe de recherche qui parlent couramment les deux langues. Les transcriptions ont été lues plusieurs fois par l’équipe parallèlement à l’enregistrement. Cela a permis d’évaluer l’exactitude de la transcription et de la traduction, et de se familiariser avec le contenu des transcriptions. Après nettoyage, les transcriptions ont été importées dans Atlas-TI version 7.5.7 pour le codage.

Le plan d’analyse a été élaboré à l’aide des thématiques de notre guide d’entretien et aussi en tenant compte des transcriptions des entrevues. Une analyse thématique inductive et déductive (17) a été effectuée pour comprendre la place de l’engagement communautaire et de la prise en compte de la détresse psychologique dans l’amélioration de la gestion des épidémies de MVE. Le corpus des données a été analysé de manière systématique par l’identification et l’examen minutieux des expressions émergentes, liées aux thèmes ayant émergé.

## Résultats

Les principales thématiques exploitées sont en lien avec le vécu des communautés face à la MVE au regard des enjeux psychologiques, leurs attitudes et perceptions en prenant en compte l’approche « Une seule santé », et la manière dont les communautés devraient être impliquées dans la gestion des urgences sanitaires, notamment la MVE. Les participants à l’étude n’avaient pas les mêmes perceptions sur la maladie : les personnes guéries l’associaient parfois à des croyances surnaturelles, tandis que le personnel soignant la considérait comme une maladie grave. Cependant, tous les participants étaient unanimes sur le besoin d’impliquer la communauté dans les stratégies de lutte, en adoptant une approche participative ascendante.

### L’expérience vécue par les communautés

Les communautés touchées par l’épidémie de MVE ont vécu des expériences douloureuses, marquées par la perte de proches. Les personnes interrogées ont souligné une méfiance envers les systèmes sanitaires, exacerbée par la présence d’expatriés et par les défaillances perçues dans la réponse à l’épidémie. Les perceptions locales, influencées par des croyances sociales et religieuses (liées au péché – entrainant la maladie et la mort) ont initialement entravé l’acceptation des mesures médicales et préventives. De plus, des accusations de clientélisme et de motivations financières (« Ebola business ») ont renforcé cette méfiance. Un survivant a témoigné de la situation en disant : « *Au lieu de s’attaquer à la maladie, ils se sont attaqués d’abord à la fortune et au luxe dans la ville* ».

Un jeune du groupe de discussion souligne : *« Mais au-delà de ça, la population aussi voyait l’argent. . . On sait que moi j’avais terminé par exemple en technique de développement. Mais on me trouve dans une structure, je commence à porter les équipements de protection individuelle pour soigner. Immédiatement la population se désengage et se dit : « Je connais ce Monsieur, il avait terminé à l’Institut du bâtiment, mais comment aujourd’hui peut-il être médecin ! ».*

La survenue de la MVE a toutefois amené un changement positif de certains comportements des communautés à savoir une amélioration des mesures d’hygiène et une gestion rationnelle des cadavres. Certains participants sont d’avis que cette maladie pourrait être facile à gérer ou éradiquer si ceux en première ligne de la riposte n’en faisaient pas un commerce sous le nom d’« Ebola business ». D’autres considèrent que les mesures de lutte sont cruciales pour aider la communauté à prévenir et limiter la propagation des épidémies.

À cet égard, un leader communautaire a partagé son observation : *« La communauté voit ces mesures comme une blague, mais quand il y a plusieurs décès, elle voit maintenant la nécessité de ces mesures ici chez nous, à Butembo. avant la survenue des décès, les personnes déchiraient les kits de lavage de mains. Mais après avoir observé plusieurs décès dans les quartiers et hôpitaux, chacun commençait à solliciter ce kit de lavage de main. »*

### Adhésion de différentes parties prenantes

Selon les participants, au début de la riposte, il y a eu plusieurs défaillances car les membres de la communauté locale n’étaient pas impliqués et aussi à cause de l’intervention de nombreux acteurs étrangers dont certains ont donné l’impression de tirer profit de la crise. Mais par la suite, les leaders communautaires et les agents de santé ont participé à la sensibilisation, amenant les gens à changer leurs comportements et attitudes à l’égard des équipes qui descendaient dans les communautés pour assurer la surveillance et le suivi des contacts.

L’éventuelle adhésion communautaire dans les trois mois suivant le début de l’épidémie a joué un grand rôle dans la surveillance. Elle a permis de créer une synergie en rapprochant toutes les couches sociales de la communauté afin de lutter efficacement contre la maladie.

### Pratiques des communautés face à la MVE

Il y a eu des divergences d’opinions sur les pratiques liées aux attitudes face à la MVE. Certains ont déclaré qu’ils gardaient leurs malades chez eux, en famille, pour avoir recours à la médecine spirituelle ou traditionnelle, La population locale ayant peur du centre de traitement Ebola qui était considéré comme un mouroir. D’autres ont relevé que le malade devait être acheminé vers une formation sanitaire pour une meilleure prise en charge.

Un leader communautaire a déclaré : *« bon, chez moi, dans la famille, personne n’avait eu Ebola ; et pour les amis qui habitaient les foyers d’Ebola, je ne cessais de les encourager à prendre le vaccin. À l’époque j’étais fiancé et ma fiancée avait reçu le vaccin grâce à nos sensibilisations. »*

### Ressenti des malades, des familles et de la communauté

Selon les informateurs clés, les formes de stigmatisation ou d’enjeux psychologiques com-prenaient principalement la peur, l’évitement, le rejet, la détresse ou l’abandon du conjoint malade, de même que le refus de réintégration dans la communauté ou de l’acceptation par la communauté.

Un membre du personnel de santé guéri d’Ebola révèle : *« Par rapport à moi et ma famille et la communauté, nous étions très stressés par cette maladie, mais quelques voisins avaient refusé catégoriquement. Avec l’appui de quelques membres de ma famille, j’ai eu la force de surmonter cette maladie. »*

### L’élaboration des mesures de lutte

Les entrevues montrent un fort consensus sur l’idée que toutes les mesures de lutte contre une épidémie doivent être élaborées avec l’implication de la communauté pour une appropriation efficace. Ces initiatives doivent tenir compte des normes socioculturelles de la population locale.

Un membre du personnel de santé a déclaré : *« c’est d’une manière participative, c’est-à-dire faire participer la population en associant ses leaders communautaires et religieux, les groupes des femmes et des jeunes dans la conception et la vulgarisation de ces mesures. »*

Un jeune a précisé : *« C’est vrai que les autorités peuvent concevoir, mais une fois la conception finie ou faite au niveau du sommet, c’est au niveau de la base qu’il y aura une difficulté à la matérialiser. On va se dire : “ça, ça concerne les autorités”. Mais quand on participe déjà à l’action ensemble, l’appropriation vient facilement. »*

### La souffrance psychologique des survivants et des communautés touchées par Ebola pendant et après la maladie

Les survivants et les communautés touchées par le virus Ebola ont subi une réelle souffrance ou traumatisme psychologique du fait de l’abandon, du rejet ou de la discrimination par les autres membres de la communauté. L’apport des leaders religieux au moyen d’un soutien psychologique a été efficace pour soulager la souffrance psychologique de ces survivants et faciliter leur réinsertion sociale et professionnelle. Parmi les guéris, certains ont perdu des membres très proches de leur famille. Les souffrances psychologiques subies ont créé une instabilité nécessitant une assistance psychologique.

Un leader communautaire a déclaré que : *« les survivants ou les communautés touchées par Ebola souffrent de blessures intenses, car ils ont perdu les membres de leur famille et d’autres familles sont en conflit à cause d’Ebola. »*

La MVE a révélé l’importance des enjeux psy-chologiques et sociaux qui constituent un frein à l’engagement des communautés dans la riposte aux flambées d’Ebola, ainsi que dans d’autres urgences sanitaires comme la Covid-19.

### Comment les communautés conçoivent l’approche « Une seule santé »

En ce qui concerne l’approche « Une seule santé » qui prône une collaboration entre les secteurs de la santé humaine, animale et environnementale, la majorité des répondants estiment qu’il est impérieux d’intégrer toutes les couches sociales de la communauté afin de faciliter l’élaboration de messages éducatifs visant à modifier les comporte-ments des populations locales. C’est une conception communautaire de l’approche « Une seule santé ».

Un leader communautaire a souligné : *« il faut impliquer toutes les couches sociales, en associant leurs leaders à l’exécution de l’action et cela, couche sociale par couche sociale (femme, jeune, groupe de pression, mutualité, etc.). »*

## Discussion

Les résultats de notre étude démontrent que les communautés doivent être impliquées dans les stratégies de réponse aux épidémies et que les aspects psychologiques tels que la peur, la stigmatisation ou la détresse dont certains membres de ces communautés sont victimes ont une influence négative sur la pleine participation des communautés dans la réponse. Au-delà de ces aspects négatifs, la communauté finit malgré tout par s’approprier la riposte et s’impliquer pour contrôler l’épidémie et sauver des vies (18). L’approche « Une seule santé » doit être privilégiée, car ces menaces touchant l’humain sont à l’interface homme, animal et environnement, mais une attention aux enjeux et aux structures des multiples secteurs impliqués pourrait devenir techniciste et bureaucratisée ; nos résultats soulignent l’importance de l’écoute et de la sensibilité aux communautés et aux personnes affectées (19,20).

La mobilisation communautaire est un des mécanismes efficaces pour les activités de promotion de la santé comme les campagnes de vaccination contre la MVE au Liberia (11). Dans un système de surveillance fonctionnel, la notification des cas doit se faire de façon régulière afin de favoriser une détection précoce. Les professionnels de santé et les agents communautaires sont formés à ce mécanisme de surveillance permettant une détection précoce d’une épidémie et une réponse rapide pour juguler le phénomène (21). Le rôle des anthropologues et des psychologues a été jugé important dans la compréhension et l’explication des cultures locales lors de ces flambées de MVE (20). La culture complexe des populations touchées par la maladie affecte la manière dont ces populations comprennent le drame qui leur arrive et leur façon de réagir ou de s’impliquer positivement ou négativement dans la gestion de la crise (22,23).

La surveillance à base communautaire constitue un atout dans la gestion des flambées de MVE avec un accent mis sur la communication et le partage d’informations entre les différents secteurs impliqués (22,24). Or, l’implication des communautés dans la gestion des épidémies de MVE est souvent tributaire des attitudes et comportements des acteurs de la riposte (25), tels les experts rendus sur le terrain, les professionnels de santé, les acteurs humanitaires.

Dans la plupart des épidémies de MVE, les survivants et leurs familles font l’objet d’une grande stigmatisation comme pour le VIH (26). Cette stigmatisation, couplée à la peur et à la détresse (27–29) peut entraver l’implication des communautés dans la gestion de la maladie (26,28,30).

Les communautés affectées par la MVE font aussi face à des problèmes de détresse psychologique liés aux conflits armés dans ces régions, comme en Sierra Leone et actuellement en RDC (31). Or, malgré les interventions importantes des organisations humanitaires et des organismes des Nations unies pour vaincre la crise de la MVE en Afrique de l’Ouest, la composante santé mentale a été jusqu’à maintenant peu considérée tant chez les patients qu’auprès des professionnels de santé (27,30). La peur qui accompagne la survenue de l’épidémie de MVE rend sa gestion difficile. De même, la faible résilience des communautés et des systèmes de santé en RDC ne permet pas non plus un contrôle rapide des flambées(7). Le cas de la Guinée en rapport avec la double « exceptionnalité » tant au niveau épidémiologique que social pourrait servir d’exemple (32,33).

Pour mettre en œuvre les recommandations issues de notre recherche, l’engagement communautaire devra être recherché dès le début de la crise par une élaboration participative des stratégies de réponse. Lorsque celles-ci sont élaborées par les autorités sans la pleine participation des populations concernées, ces dernières se sentent non considérées et nous assistons à une faible ou à un manque d’implication des communautés (1). C’est pourquoi, avec notre expérience en tant qu’expert et coordonnateur de la réponse dans les multiples épidémies de MVE en Guinée et en RDC, il nous apparait dès lors comme une évidence de travailler non pas avec côtés des communautés, mais avec ces dernières. Les approches de la participation communautaire devront être revisitées pour rencontrer les préoccupations et les aspirations de la communauté. L’expérience de la Guinée a montré que c’était quand les résistances devenaient de plus en plus persistantes et explosives que les acteurs de la riposte décidaient d’impliquer les leaders (34).

Les représentations sociales ou religieuses de la maladie ont un rôle majeur dans la perception de la maladie et par ce fait, influencent fortement les perceptions positives ou négatives des communautés envers les interventions de santé publique. La maladie à virus Ebola tout comme la récente pandémie à Covid-19 sont encore des maladies avec une forte connotation spirituelle dans les pays africains malgré la disponibilité des vaccins (35), car l’immunité conférée par ces vaccins n’est pas connue et des rechutes ou réinfections ont été constatées.

Les membres des communautés touchées par la MVE ainsi que les professionnels de la santé et les experts reconnaissent le fardeau de la souffrance tant humaine que psychologique des survivants (36). L’humanisation des soins pour ces épidémies a contribué largement à permettre le recours aux soins de santé moderne dans les établissements de soins, car les communautés pouvaient accéder aux malades et même leur apporter un soutien moral et nutritionnel. Il faut développer alors des stratégies ou des approches innovantes pour arriver à une pleine implication communautaire. Le rôle de la communication est essentiel notamment dans une (37) communication sur les risques et l’engagement des communautés. L’analyse du non-engagement des communautés ne devrait pas se limiter seulement aux infrastructures sanitaires ou au faible niveau du système de santé ; la prise en compte des pratiques culturelles, des problèmes sociaux et économiques ne doit pas être occultée (38). De par notre expérience de terrain, il est important d’impliquer toutes les couches et expertises, d’où l’importance de privilégier l’approche « une seule santé » où tous les secteurs pertinents de la lutte participent à la riposte avec une collaboration multi et intersectorielle.

Le nouveau modèle de gestion des incidents proposé par les CDC (Centres américains pour le contrôle et la gestion des maladies), le gestionnaire de l’incident appelé « incident manager » (39) devrait être un expert du lieu malgré l’obtention d’appuis venant d’un autre niveau. Il arrive souvent que la participation des experts dits « étrangers » – du fait qu’ils ne sont pas de ces communautés affectées – soit souvent mal perçue par ces dernières, car ces « étrangers», mêmes s’ils sont des nationaux, viennent leur « voler du travail » et s’enrichir sur leur dos, reproduisant ainsi une sorte d’« Ebola business » déjà décrié.

Il est important dans chaque contexte d’identifier les mesures ou pratiques communautaires pouvant être considérées comme facilitatrices de l’engagement communautaire et les mesures ou pratiques dites barrières à l’engagement dans le contexte de la Sierra Leone lors de l’épidémie de 2015 (40).

## Conclusion

Une gestion efficace et efficiente des épidémies ne peut se réaliser sans l’implication active des communautés. Les enjeux psychologiques tels que la peur, la détresse et la stigmatisation, pouvant entraver la pleine participation des populations, doivent être pris en compte. Ces facteurs ne sont pas seulement des obstacles à surmonter, mais aussi des aspects cruciaux du bien-être humain méritant attention. Forts de notre expérience d’acteur de terrain, nous soulignons l’importance d’une approche collaborative impliquant tous les secteurs dans la réponse aux épidémies. L’approche « Une seule santé » doit être encouragée et privilégiée. Le cycle de gestion des épidémies, notamment celles d’Ebola et même de Covid-19, passe par une phase de méfiance et de résistance, puis d’acceptation et d’engagement des communautés. Les décideurs et professionnels de santé doivent être attentifs aux perceptions et aux craintes des communautés affectées pour mieux les accompagner. Nous recommandons un modèle innovant de gestion des épidémies et urgences de santé publique intégrant les déterminants clés de l’engagement communautaire, notamment l’approche « Une seule santé » et la prise en compte de la détresse psychologique, souvent négligée dans les stratégies de réponse. La peur et la détresse sont des facteurs à prendre en compte lors de l’élaboration de stratégies innovantes de lutte.
